# New genera, a new species, and a key to the genera of Ashieldophyinae (Acari, Eriophyoidea) from India

**DOI:** 10.3897/zookeys.843.29078

**Published:** 2019-05-09

**Authors:** Samiran Chakrabarti, Ramkrishna Pandit, Surajit Sur

**Affiliations:** 1 Post-Graduate Department of Zoology, Vidyasagar College, 39 Sankar Ghosh Lane, Kolkata 700006, India Department of Zoology, Vidyasagar College Kolkata India; 2 Department of Zoology, Mahadevananda Mahavidyalaya, Barrackpore, Kolkata 700120, India Department of Zoology, Mahadevananda Mahavidyalaya Kolkata India

**Keywords:** *
Ashieldophyes
*, *Brevishieldophyes* gen. n., comparison, descriptions, *Mesoshieldophyes* gen. n., *Mesoshieldophyesvarecae* sp. n., *Brevishieldophyesglochidionae* comb. n.

## Abstract

Two new genera, *Brevishieldophyes* Chakrabarti & Pandit, **gen**. **n**. and *Mesoshieldophyes* Chakrabarti & Pandit, **gen**. **n**., and a new species *Mesoshieldophyesvarecae* Chakrabarti & Pandit, **sp**. **n**. are described. These mites are leaf vagrants. The morphological characters of the afore-mentioned genera and those of *Ashieldophyes* Mohanasundaram are compared. A key for separating the genera within the subfamily Ashieldophyinae is provided. The diagnostic characters of the subfamily Ashieldophyinae are also revised.

## Introduction

*Ashieldophyespennadamensis* Mohanasundaram, 1984, infesting *Caseariatomentosa* Roxb. (Salicaceae) from near the Pennadam Sugar Factory, Arcot district, Tamil Nadu, south India, was the type species for the genus *Ashieldophyes* Mohanasundaram within the new family Ashieldophyidae Mohanasundaram. Later, the family Ashieldophyidae was made one of the subfamilies (Ashieldophyinae) of the EriophyidaeNalepa (1898)because a small prodorsal shield was actually observed on the propodosoma of the mite (Amrine and Stasny 1994; Amrine 1996; Amrine et al. 2003). A second species, *Ashieldophyesglochidionae* Chakrabarti & Pandit, 2009, infesting *Glochidionmultiloculare* (Rottler ex Willd.) Voigt (Phyllanthaceae) from Lataguri Forest, Jalpaiguri, West Bengal, was described in this taxon.

During periodical samplings for exploration of eriophyoid mite diversity in West Bengal & Assam, further samples of eriophyoids infesting *Caseariavareca* Roxb. and *C.glomerata* Roxb. were collected. Examination of those specimens allowed establishing two new genera, *Mesoshieldophyes* Chakrabarti & Pandit, gen. n. for accommodating *Mesoshieldophyesvarecae* Chakrabarti & Pandit, sp. n. and *Brevishieldophyes* Chakrabarti & Pandit, gen. n. for reassigning *A.glochidionae* in the Ashieldophyinae.

## Materials and methods

Eriophyoid mites were collected and studied as described by Chakrabarti et al. (2017). The terminology and classification given by Lindquist (1996) and Amrine et al. (2003), respectively are followed here. The specimens were examined with a phase contrast Leica DM3000 microscope and photographs were taken with Leica DFC295 camera. All measurements were made following Amrine and Manson (1996) and de Lillo et al. (2010), and are given in micrometres (µm). Measurements and means are rounded off to the nearest integer and refer to the length of the morphological characters unless specified otherwise. Drawings were made following de Lillo et al. (2010) and Amrine et al. (2003). In the text, measurements of the holotype are followed by the range of measurements of the paratypes plus holotype given in parentheses. All type specimens are now deposited in the collection of the Post-Graduate Department of Zoology, Vidyasagar College, Kolkata 700006, India. After publication, holotypes and paratypes will be deposited in public institutions: one slide with paratypes of each species will be deposited to the National Pusa Collection, Indian Agricultural Research Institute, New Delhi; the holotype and the remaining paratypes will be deposited in the National Zoological Collection, Zoological Survey of India, Kolkata.

## Taxonomy

### Ashieldophyes

Taxon classificationAnimaliaTrombidiformesEriophyidae

Mohanasundaram, 1984

http://zoobank.org/9A73DBEB-3C29-44D3-89DF-B349D0C8F895

#### Diagnosis.

Prodorsal shield small and oval shaped; scapular tubercles absent but with very short scapular setae *sc*, placed on lateral margins, directed laterally; pedipalp genual setae *d* present and simple; femoral setae *bv* of leg I present; genual setae *l*″ of leg II present; coxae with setae *1b*; female genitalia located between coxae II; genital cover flap lacks ridges.

#### Type species.

*Ashieldophyespennadamensis* Mohanasundaram, 1984.

#### Remarks.

*Ashieldophyes* Mohanasundaram, 1984, *Brevishieldophyes* Chakrabarti & Pandit, gen. n., and *Mesoshieldophyes* Chakrabarti & Pandit, gen. n. belong to the subfamily Ashieldophyinae of family Eriophyidae in having small or moderately developed shield, lacking opisthosomal setae *d* and *e*, coxae widely separated anteriorly, female genitalia appressed to the coxae and with a triangular cover flap. These three genera can easily be separated by the characters given in Table 1 and in the key provided below. The genus is monotypic.

**Table 1.** Data set for some morphological characters of *Ashieldophyes*, *Brevishieldophyes*, and *Mesoshieldophyes*.

**Characters**	*** Ashieldophyes ***	*** Brevishieldophyes ***	*** Mesoshieldophyes ***
Body	Vermiform	Fusiform	Fusiform
Pedipalp genual setae *d*	Present	Present	Absent
Prodorsal shield	Small, oval shaped	Small, sickle shaped	Semi-circular, comparatively larger.
Scapular tubercles	Absent	Absent	Absent
Scapular setae *sc*	Present (very short)	Absent	Absent
Femoral setae *bv* on leg I	Present	Present	Absent
Solenidion *ω*	Blunt	Knobbed	knobbed
genual seta *l*″ on Leg II	Present	Present	Absent
Dorsal and ventral semiannuli	Equal number, smooth	Equal number, smooth	Equal number, granular
Seta *1b*	Present	Absent	Present

### Ashieldophyes pennadamensis

Taxon classificationAnimaliaTrombidiformesEriophyidae

Mohanasundaram, 1984

http://zoobank.org/D84AC07F-CD6A-42E1-9E42-EA6782545674

Fig. 1 AD1, CG1

Ashieldophyes pennadamensis Mohanasundaram, 1984, Oriental Insects, 18: 251–252.

#### Diagnosis.

Body vermiform; pedipalp genual seta *d* present; prodorsal shield small and oval; scapular tubercles absent but with very short setae *sc*; legs with all usual setae; solenidion *ω* blunt; opisthosoma with equal number of smooth dorsal and ventral semiannuli; setae *1b* present.

#### Description.

Female (n = 20). Body vermiform, brown colour in life, dorso-ventrally flattened; 250 (175–250) and 40 (39–46) wide. **Gnathosoma** 15 (14–15) projecting obliquely down-curved, dorsal pedipalp genual setae *d* 1 (1–2); chelicerae 13 (13–15). **Prodorsal shield** small, oval-shaped, without lobe, 9 (9–10) and 23 (22–23) wide, lacking scapular tubercles but with very short scapular setae *sc*, placed on lateral margin and directed laterally. **Leg I** from base of trochanter 20 (20–21), femur 7 (7–8), femoral setae *bv* 7 (7–8), genu 3 (2–3), genual setae *l*″ 20 (21–23), tibia 5 (3–5), tibial setae *l*′ 15 (12–15), tarsus 5 (3–5), tarsal setae *ft*′ and *ft*″ both 12 (10–12), solenidion *ω* 4 (3–4), straight and blunt; empodium *em* simple, 4-rayed; setae *u*′ 2 (2–3). **Leg II** from base of trochanter 18 (18–20); femur 6 (5–6), femoral setae *bv* 5 (5–6), genu 2 (2–3), genual setae *lʺ* 23 (20–23), tibia 3 (3–4), tibial setae *lʹ* absent, tarsus 4 (3–4), solenidion *ω* 8 (7–8), straight and blunt; empodium *em* simple, 4-rayed; tarsal setae *ftʹ* 8 (8–10) and *ftʺ* 12 (10–12), setae *uʹ* 2 (2–3). **Coxigenital area** smooth; broadly joined, sternal line absent, coxa I widely separate, setae *1b* 2 (2–3) and 5 (5–6) apart, setae *1a* 8 (8–9) and 7 (7–8) apart, setae *2a* 13 (13–15) and 18 (18–20) apart. **Opisthosoma** dorsally flat, smooth, with equal number of dorsal and ventral semiannuli, 21 (20–21); setae *c2* 10 (7–11) on ventral semiannulus 2 (2–3), setae *d* and *e* absent, setae *f* 14 (12–15) on ventral semiannulus 7 (6–7) from rear margin; setae *h1* absent, setae *h2* 12 (12–14). **Genital cover flap** 10 (9–11) and 16 (17–18) wide, triangular and smooth; setae *3a* 8 (6–8). **Internal genitalia** apodeme short, spermathecae rounded with short funnel-like spermathecal tubes.

**Figure 1.**
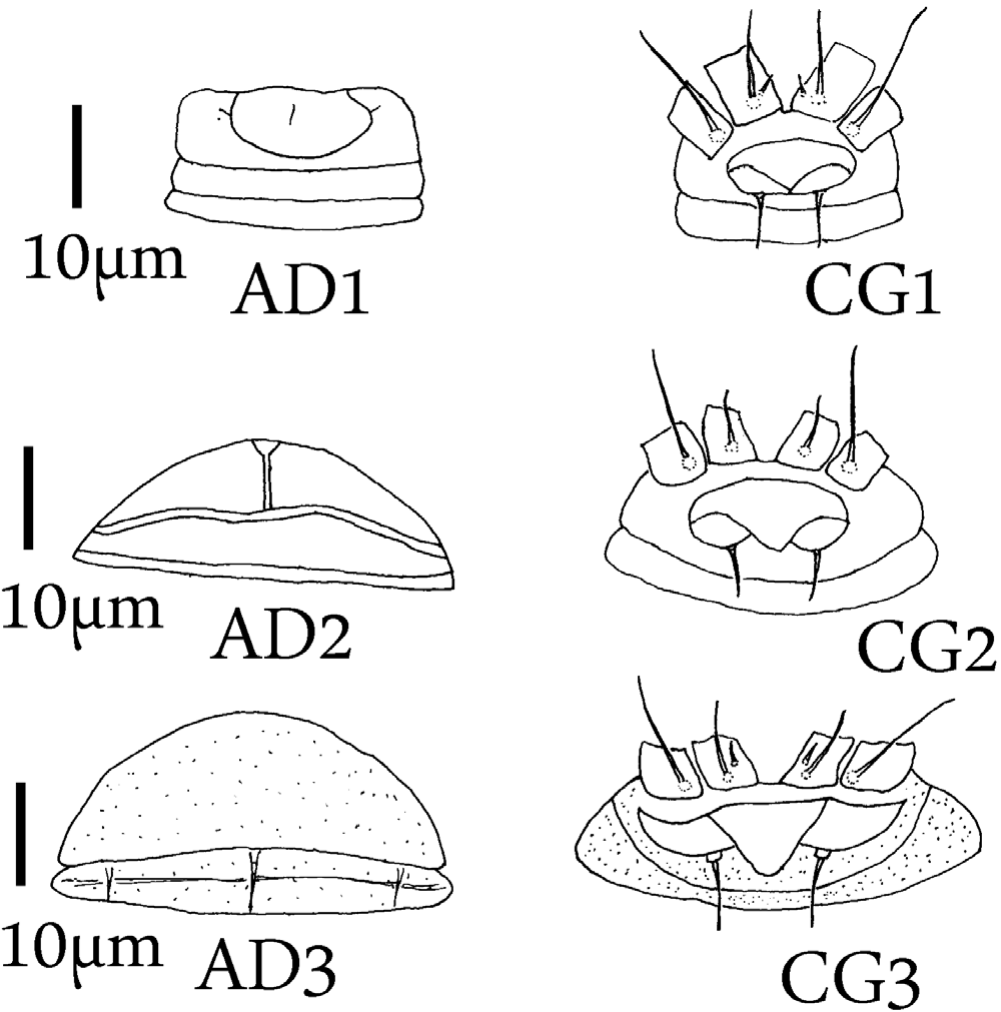
Female: Antero-dorsal region **AD** and coxigenital region **CG AD1** and **CG1** of *Ashieldophyespennadamensis***AD2** and **CG2** of *Brevishieldophyesglochidionae***AD3** and **CG3** of *Mesoshieldophyesvarecae*.

#### Specimens examined.

India: Tamil Nadu: South Arcot District, near Pennadam Sugar Factory, 16.VIII.1981, 2 females from *C.tomentosa*, coll. M. Mohanasundaram, coll. no. 427 (TNAU); West Bengal: North 24-Parganas, Madral, 12.VIII.2005, many females and nymphs from *C.tomentosa*, coll. R Pandit, coll. nos. 1182–1184/19/2005.

#### Distribution.

India: Tamil Nadu & West Bengal.

#### Relation to the host plant.

The mites inhabit the under surface of leaves as vagrants without showing symptoms of damage to the host plant.

#### Remarks.

This species is so far known only from its type locality and here reported for the first time from West Bengal.

### Brevishieldophyes

Taxon classificationAnimaliaTrombidiformesEriophyidae

Chakrabarti & Pandit gen. n.

http://zoobank.org/16667767-867C-4329-A2F9-CF791F511F0B

#### Diagnosis.

Body fusiform, dorso-ventrally flattened. Gnathosoma short, downwardly curved, cheliceral stylet short; prodorsal shield small and sickle-shaped; scapular tubercles and scapular setae *sc* lacking; pedipalp genual setae *d* present and simple; coxae without setae *1b*; femoral setae *bv* of leg I present; genual setae *l*″ of leg II present; empodium simple; female genitalia appressed to the coxae, genital cover flap smooth, triangular, located between coxae II; apodeme normal in length.

#### Type species.

*Ashieldophyesglochidionae* Chakrabarti & Pandit, 2009.

The genus is monotypic.

#### Etymology.

*Brevi* derived from the adjective Latin word *brevis* meaning short (in relation to prodorsal shield) and *ophyes* derived from *eriophyes* meaning *erion* = wool + phyes = a grower/maker.

#### Gender.

Masculine.

### Brevishieldophyes glochidionae

Taxon classificationAnimaliaTrombidiformesEriophyidae

(Chakrabarti & Pandit, 2009) comb. n.

http://zoobank.org/33D1FFB7-997E-495E-AD21-EF1EDB27CB2B

Ashieldophyes glochidionae Chakrabarti & Pandit, 2009, International Journal of Acarology, 15:163–164.

#### Diagnosis.

Body fusiform; pedipalp genual seta *d* present; prodorsal shield small and sickle shaped; scapular setae *sc* absent; legs with all usual setae; solenidion *ω* knobbed; opisthosoma with equal number of smooth dorsal and ventral semiannuli; setae *1b* absent.

#### Specimens examined.

India: West Bengal: Jalpaiguri, Lataguri forest, 15.X.2004, many females and nymphs from *G.multiloculare*, coll. R Pandit. coll. nos. 1275–1279/45/2004. Type material of *A.glochidionae* Meghalaya: Burnihat, 18.X.1985, many females and nymphs, from *C.glomerata*, coll. B Das, coll. nos. 967–971/61/1985.

#### Distribution.

India: West Bengal & Meghalaya.

#### Relation to the host plant.

The mites inhabit the under surface of leaves as vagrants without showing symptoms of damage to the host plant.

#### Remarks.

The original report of *B.glochidionae* from *G.multiloculare* needs further confirmation because this mite species and other two Ashieldophyinae have been collected subsequently from plants of the genus *Casearia* (Salicaceae).

### Mesoshieldophyes

Taxon classificationAnimaliaTrombidiformesEriophyidae

Chakrabarti & Pandit gen. n.

http://zoobank.org/7227C05D-E910-44EE-AC65-49CACF572032

#### Diagnosis.

Body fusiform, dorso-ventrally flattened. Gnathosoma short, obliquely down-curved, cheliceral stylet short; pedipalp genual setae *d* absent; prodorsal shield semi-circular without any lobe, lacking scapular tubercles and scapular setae *sc*; femoral setae *bv* of leg I and genual setae *l*″ of leg II absent; coxae with setae *1b*; dorsal and ventral semiannuli with granules; female genitalia appressed to the coxae; genital cover flap triangular and smooth; empodium simple; apodeme short in length.

#### Type species.

*Mesoshieldophyesvarecae* Chakrabarti & Pandit, sp. n.

This genus is monotypic.

#### Etymology.

The genus name *Mesoshieldophyes* is derived from *meso* = middle, referring to the medium size of prodorsal shield and *phyes* derived from *eriophyes* meaning *erion* = wool + *phyes*, a grower/maker.

#### Gender.

Masculine.

#### Remarks.

The size of the prodorsal shield in this genus is larger than that in the other two genera of this subfamily.

### Mesoshieldophyes varecae

Taxon classificationAnimaliaTrombidiformesEriophyidae

Chakrabarti & Pandit sp. n.

http://zoobank.org/E19A73E2-8A4C-4ABD-8C2F-4FC2DDFBC135

#### Diagnosis.

Body fusiform; pedipalp genual seta *d* absent; prodorsal shield semi-circular; scapular setae *sc* absent; femoral setae *bv* on leg I absent; genual setae *lʺ* on leg II absent; solenidion *ω* knobbed; opisthosoma with equal number of granulated dorsal and ventral semiannuli; setae *1b* present.

#### Description.

Female (n=12). Body fusiform, yellow colour in life, dorso-ventrally flattened; 140 (120–145) and 50 (45–50) wide. **Gnathosoma** 15 (14–15) projecting obliquely down-wards, dorsal pedipalp genual setae *d* absent, setae *ep* 1 (1–2); chelicerae 13 (13–15). **Prodorsal shield** semicircular, without lobe, 14 (18–20) and 43 (40–43) wide with granules, lacking scapular tubercles and setae *sc.***Leg I** from base of trochanter 20 (20–21), femur 7 (7–8), femoral setae *bv* absent, genu 3 (2–3), genual setae *l*″ 20 (21–23), tibia 4 (3–4), tibial setae *l*′ 10 (10–12), tarsus 5 (3–5), tarsal setae *ft*′ and *ft*″ both 12 (10–12), solenidion *ω* 4 (3–4), straight and knobbed; empodium *em* 4 (4–5), simple, 4-rayed; setae *u*′ 2 (2–3). **Leg II** from base of trochanter 18 (18–20); femur 6 (5–6), femoral setae *bv 5* (5–6), genu 2 (2–3), genual setae *l*″ absent, tibia 3 (3–4), tibial setae *l*′ absent, tarsus 4 (3–4), tarsal setae *ft*′ 8 (8–10), *ft*″ 12 (10–12); solenidion *ω* 8 (7–8), straight and knobbed; empodium *em* 4 (4–5), simple, 4-rayed; setae *u*′ 2 (2–3). **Coxigenital area** smooth; sternal line absent, coxae widely separated, setae *1b* 2 (2–3) and 5 (5–6) apart, setae *1a* 8 (8–9) and 7 (7–8) apart, setae *2a* 13 (13–15) and 18 (18–20) apart. **Opisthosoma** dorsally flat, with equal number of dorsal and ventral semiannuli, 21 (20–21), both dorsal and ventral semiannuli ornamented with fine granules; setae *c2* 10 (7–11) on ventral semiannulus 2 (2–3), setae *d* and *e* absent, setae *f* 14 (12–15) on ventral semiannulus 7 (6–7) from rear margin; setae *h1* absent, setae *h2* 12 (12–14). **Genital cover flap** 6 (5–6) and 16 (17–18) wide, triangular and smooth; setae *3a* 7 (6–7). **Internal genitalia** apodeme short, spermathecae globose with short, funnel-like spermathecal tubes.

**Figure 2.**
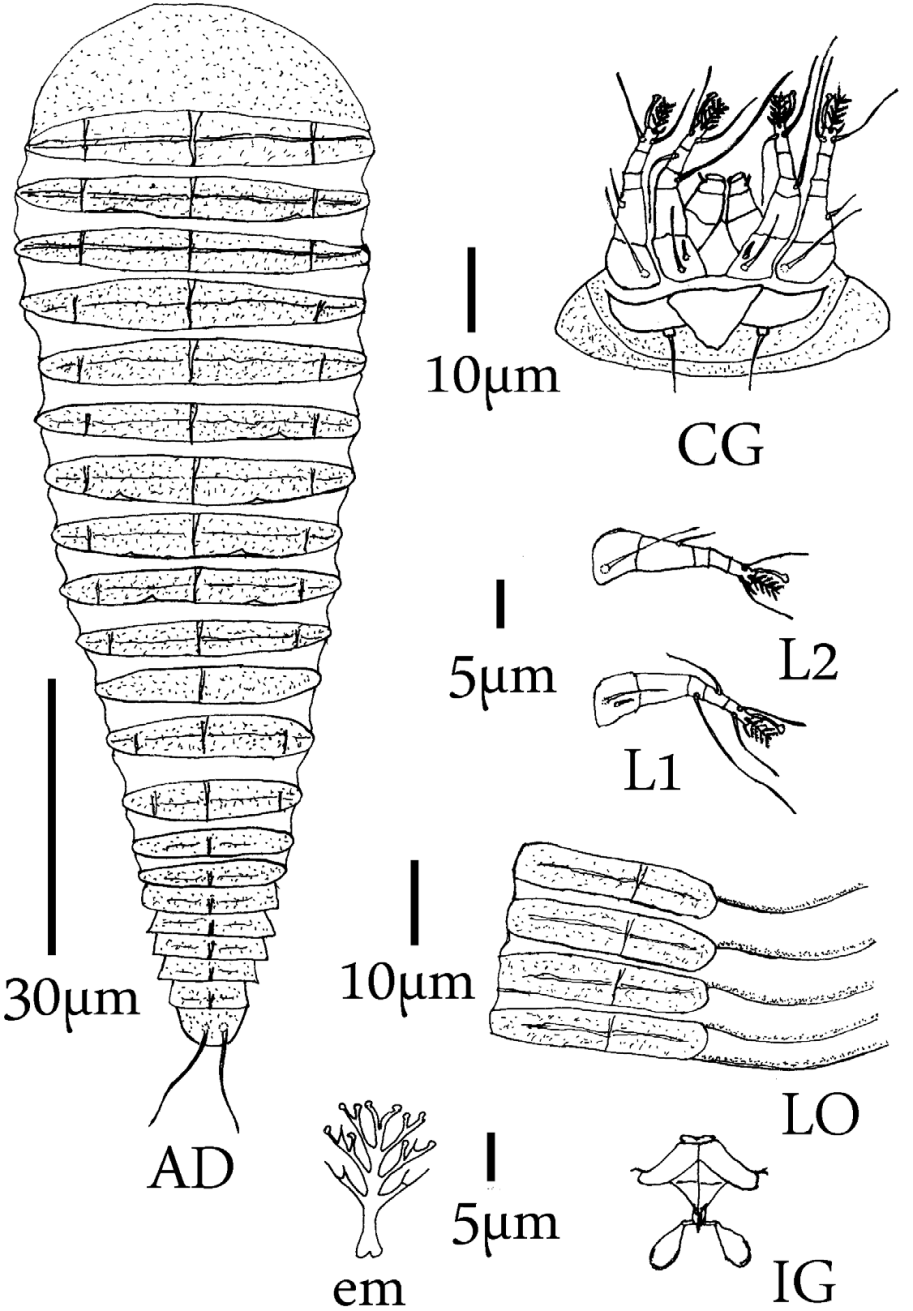
*Mesoshieldophyesvarecae*, Female. Abbreviations **CG** coxigenital region **D** dorsal view of body; **em** empodium **IG** Internal genitalia **LO** Dorsal and ventral annuli.

**Figure 3.**
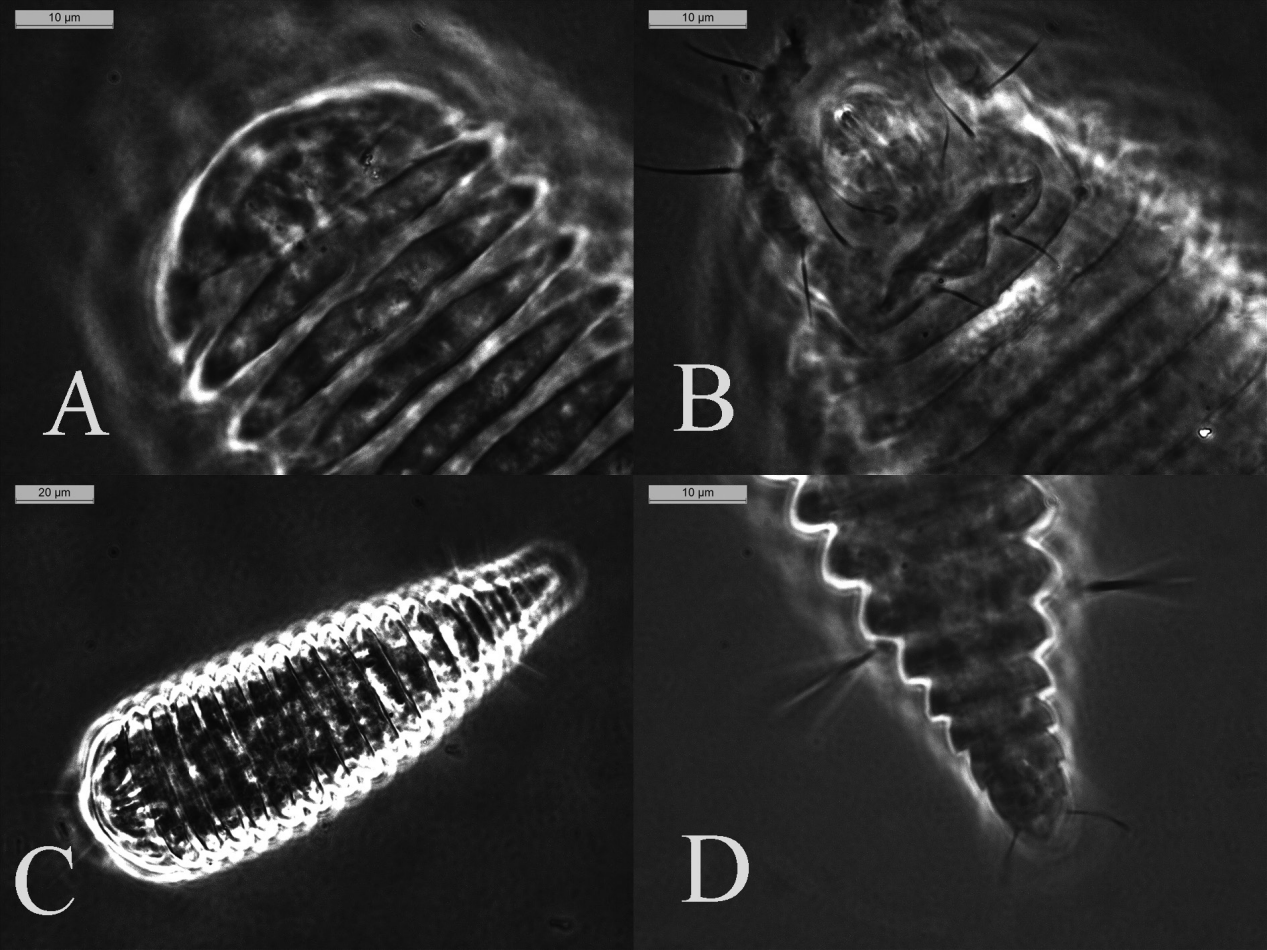
*Mesoshieldophyesvarecae*, Female **A** prodorsal shield with dorsal annuli **B** genital cover flap **C** entire dorsal body **D** posterior part of opisthosoma with setae *f* and *h2*.

Male. Not observed.

#### Type host plant.

*Caseariavareca* Roxb. (Salicaceae).

#### Relation to the host plant.

The mites inhabit the under surface of leaves as vagrants without showing symptoms of damage to the host plant.

#### Type locality.

India: West Bengal: Darjeeling, Bengdubi Forest (26°42′30.1″N, 88°25′36.7″E), 163 m above sea level, 03.II.2015, coll. S Chakrabarti, R Pandit, S Sarkar.

#### Type material.

Holotype: female marked on slide (no. 1294/N11/2015); paratypes: 2 females on slide bearing holotype and 36 females, larvae and nymphs on 10 slides (nos. 1295–1304/N11/2015).

#### Etymology.

The species name *varecae* is from the specific designation of the host plant in the genitive case.

### Key to the genera of subfamily Ashieldophyinae

**Table:** 

1	Body vermiform, scapular setae *sc* present; femoral setae *bv* on leg I and genual setae *l*” on leg II present, on Salicaceae	***Ashieldophyes* Mohanasundaram, 1984**
–	Body fusiform, scapular setae *sc* lacking	**2**
2	Prodorsal shield small, sickle shaped; coxal setae *1b* lacking; femoral setae *bv* on leg I and genual setae *lʺ* on leg II present, on Salicaceae & Phyllanthaceae	***Brevishieldophyes* Chakrabarti & Pandit, gen. n.**
–	Prodorsal shield moderate, semicircular; coxal setae *1b* present; femoral setae *bv* of leg I and genual setae *l*″ of leg II lacking, on Salicaceae	***Mesoshieldophyes* Chakrabarti & Pandit, gen. n.**

### Subfamily Ashieldophyinae Mohanasundaram (1984)

**Diagnosis.** Prodorsal shield poorly developed to moderately developed, lacking scapular tubercles, scapular setae *sc* absent but if present very short; sternal line absent; coxae widely separated anteriorly; legs with all segments, setae *bv* on leg I and genual setae *lʺ* on leg II may or may not be present; opisthosoma lacking setae *d* and *e* but *c2* and *f* present; genitalia appressed to the coxae, genital cover flap triangular; genital apodeme curved and abbreviated and spermathecae globose with short spermathecal tubes.

## Supplementary Material

XML Treatment for Ashieldophyes

XML Treatment for Ashieldophyes pennadamensis

XML Treatment for Brevishieldophyes

XML Treatment for Brevishieldophyes glochidionae

XML Treatment for Mesoshieldophyes

XML Treatment for Mesoshieldophyes varecae

## Supplementary materials

Supplementary material 1 COL Data type: image This dataset is made available under the Open Database License (http://opendatacommons.org/licenses/odbl/1.0/). The Open Database License (ODbL) is a license agreement intended to allow users to freely share, modify, and use this Dataset while maintaining this same freedom for others, provided that the original source and author(s) are credited.Samiran Chakrabarti, Ramkrishna Pandit, Surajit Sur

Supplementary material 2 Morphological images 1 Data type: image This dataset is made available under the Open Database License (http://opendatacommons.org/licenses/odbl/1.0/). The Open Database License (ODbL) is a license agreement intended to allow users to freely share, modify, and use this Dataset while maintaining this same freedom for others, provided that the original source and author(s) are credited.Samiran Chakrabarti, Ramkrishna Pandit, Surajit Sur

Supplementary material 3 Morphological images 2 Data type: image This dataset is made available under the Open Database License (http://opendatacommons.org/licenses/odbl/1.0/). The Open Database License (ODbL) is a license agreement intended to allow users to freely share, modify, and use this Dataset while maintaining this same freedom for others, provided that the original source and author(s) are credited.Samiran Chakrabarti, Ramkrishna Pandit, Surajit Sur
