# Supplementary figures and images for: New genera, a new species, and a key to the genera of Ashieldophyinae (Acari, Eriophyoidea) from India
Source: Zookeys. 2019 May 9;843:39–49. doi: 10.3897/zookeys.843.29078 (PMC6522452; doi:10.3897/zookeys.843.29078)

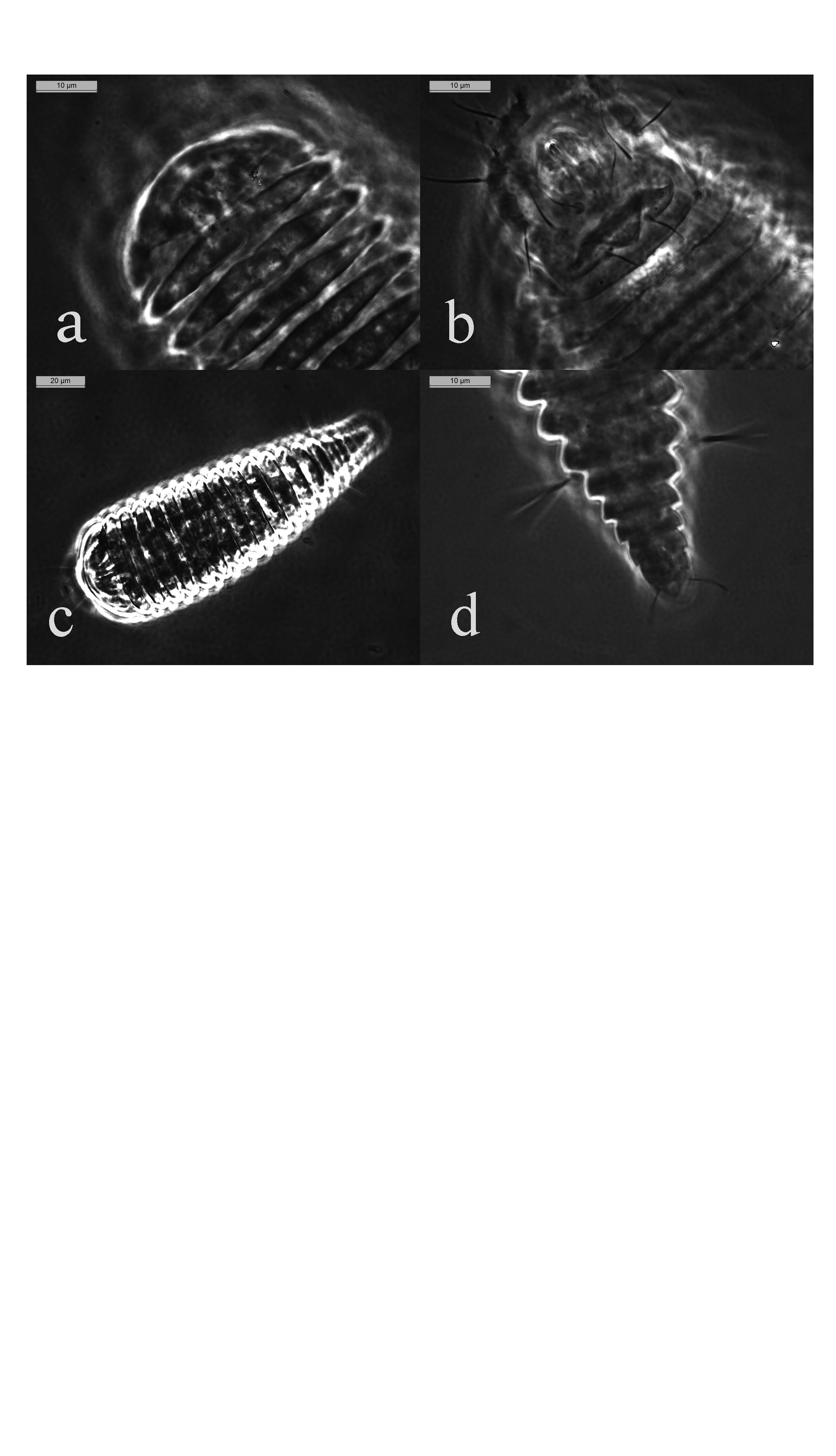

Supplement: Supplementary material 1 [file zookeys-843-039-s001.tif]

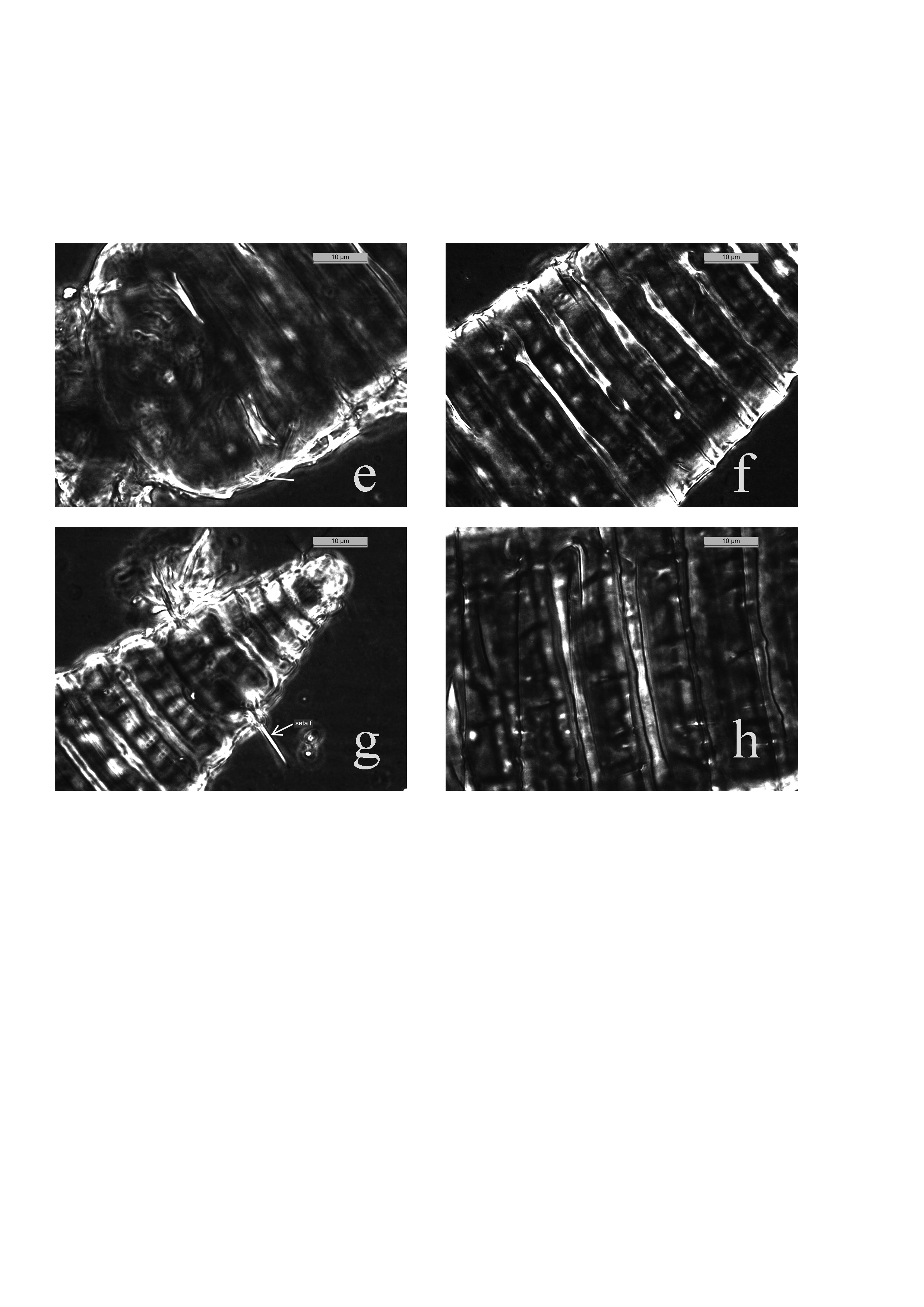

Supplement: Supplementary material 2 [file zookeys-843-039-s002.tif]

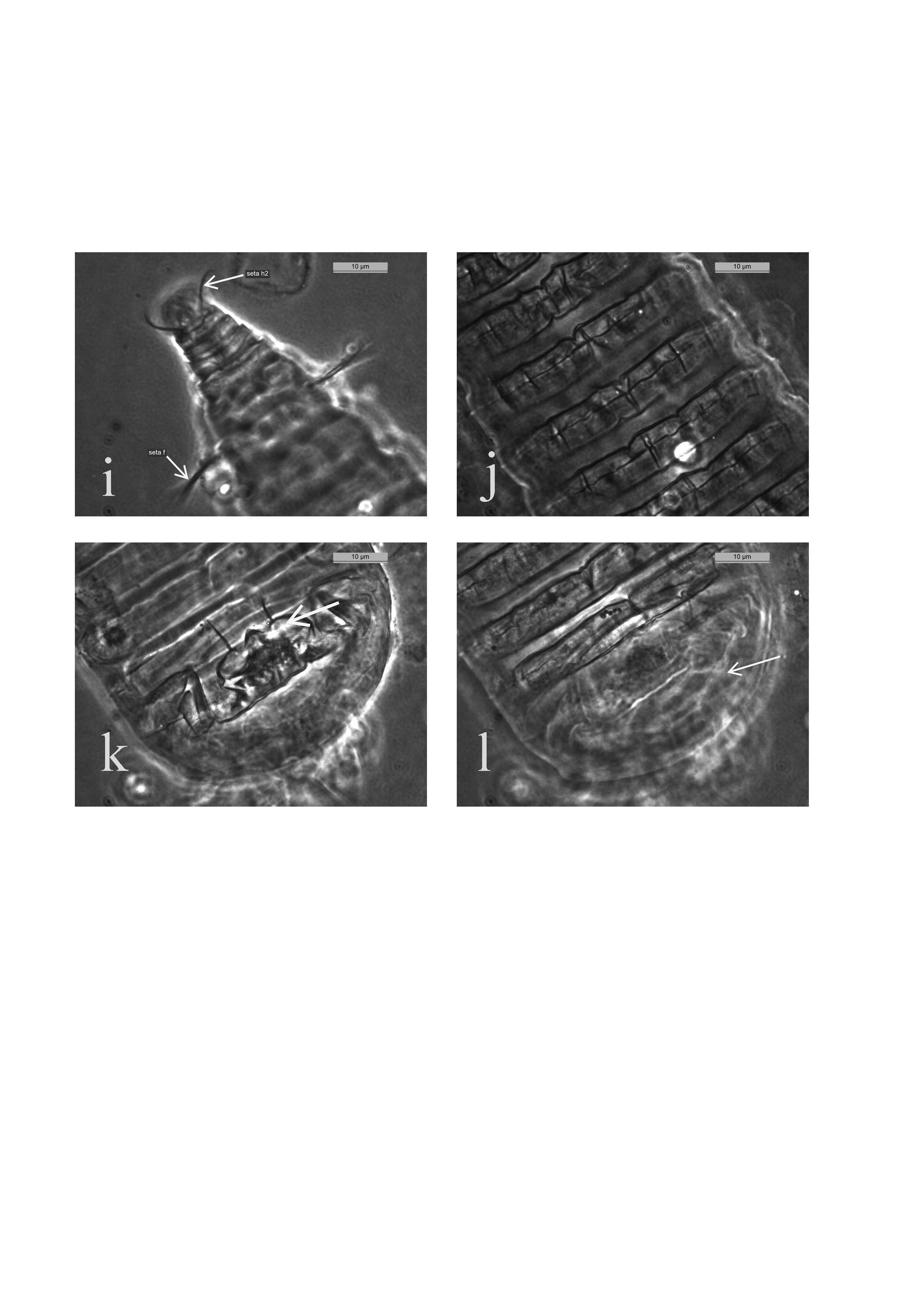

Supplement: Supplementary material 3 [file zookeys-843-039-s003.tif]
